# Optimization of Recycled Fine Aggregate Content for All-Solid-Waste-Based Flowable Solidified Soil: Performance and Microstructure

**DOI:** 10.3390/ma19173638

**Published:** 2026-08-27

**Authors:** Anhui Wang, Liwei Ju, Jiaojiao Ni, Lili Li, Enze Zhen

**Affiliations:** 1 School of Architecture and Civil Engineering, and Anhui Institute for the Preservation and Inheritance of Huizhou-Style Architecture, Huangshan University, Huangshan 245041, China; 2 China Construction Industrial and Energy Engineering Group Co., Ltd., Nanjing 210023, China; 3 Beijing Tianrun Construction Co., Ltd., Beijing 100076, China; 4 School of Civil Engineering and Architecture, Jiangsu University of Science and Technology, Zhenjiang 212000, China

**Keywords:** recycled fine aggregate, flowable solidified soil, performance optimization, durability, microstructure

## Abstract

To promote the high-value utilization of construction and industrial solid wastes, this study prepared an all-solid-waste-based flowable solidified soil (FSS) using soft clay and recycled fine aggregate (RFA) as the main constituents. The binder system comprised ground-granulated blast-furnace slag (GGBS), carbide slag (CS), and desulfurization gypsum (DG), while fly ash (FA) was incorporated to improve workability. The primary objective was to identify an appropriate RFA content for this FSS system through a combined evaluation of workability, mechanical performance, durability, and microstructural characteristics. The results showed that increasing the RFA content increased flowability and shortened the setting time. Unconfined compressive strength (UCS) and ultrasonic pulse velocity (UPV) both increased initially and then decreased as the RFA content increased, and relatively favorable mechanical performance was observed at RFA contents of 40–60%. In the durability tests, the mixture containing 40% RFA exhibited the lowest mass loss and UCS loss after both wetting–drying and freeze–thaw cycles within the investigated range. X-ray diffraction (XRD) and scanning electron microscopy (SEM) analyses suggested that a moderate RFA content was associated with the development of C-(A)-S-H-gel-related phases and ettringite (AFt), together with a denser and more continuous microstructure. The improved strength and durability at moderate RFA contents were therefore interpreted as the combined results of hydration-product development and the physical skeleton effect provided by RFA. By contrast, the performance decline at excessive RFA contents appeared to be related to a less favorable internal structure, as indicated by SEM observations. Overall, when workability, mechanical performance, durability, and microstructural observations are considered together, 40% RFA is recommended as the most suitable content for the material system and test conditions investigated in this study. These findings demonstrate the potential of RFA to regulate the performance of all-solid-waste-based FSS and to improve the resource efficiency of multiple solid-waste streams.

## 1. Introduction

With the rapid pace of urbanization in China, the annual generation of excavated soil and waste concrete has reached several billion tons [1,2]. The large-scale accumulation of these wastes not only occupies valuable land resources but also poses environmental risks [3]. Meanwhile, backfilling in foundation pits and utility trenches still relies heavily on traditional binders such as cement and lime, whose production is associated with high energy consumption and substantial carbon emissions [4]. Under these dual pressures, the efficient and low-carbon recycling of excavated soil and construction waste has become an important issue in geotechnical engineering and construction materials [5].

Flowable solidified soil (FSS) provides a practical route for addressing this issue [6,7,8]. In this technology, excavated soil is mixed with a binder and water to form a slurry with good flowability and self-compacting ability. After placement and curing, the slurry hardens into a material with adequate compressive strength for backfilling applications. As a backfill material, FSS offers several advantages, including ease of construction, pumpability, no need for vibration, and adaptability to confined construction spaces [9,10]. In recent years, to reduce dependence on cement and lime, researchers have explored the use of industrial solid wastes such as GGBS, steel slag, DG, and CS as alternative stabilizing agents [11,12,13]. Under alkaline activation, these materials can generate hydration products such as C-(A)-S-H gel and AFt, thereby stabilizing soil while reducing both material cost and carbon emissions [14,15]. However, the early hydration of solid-waste-based binders in FSS is generally slower than that of conventional cementitious systems, and problems such as long setting times, low early strength, and relatively porous internal structures remain unresolved [16,17]. Improving the mechanical performance and long-term durability of all-solid-waste-based FSS while maintaining adequate flowability therefore remains an important research objective.

Recycled fine aggregate (RFA), produced from waste concrete and brick debris through crushing and sieving, is widely available and relatively inexpensive [18]. Previous studies have shown that RFA can act as a physical skeleton in solidified soil systems [19]. At an appropriate content, it may improve particle gradation, enhance matrix compactness, and increase mechanical strength [20]. In addition, the residual cement mortar attached to the RFA surface contains incompletely hydrated clinker minerals, which may undergo secondary hydration under alkaline conditions and generate additional cementitious products [21,22]. Despite these potential advantages, existing research has focused mainly on the use of RFA in concrete [21], mortar [23,24], and conventional stabilized soils [19,20,25], whereas its role in all-solid-waste-based FSS remains insufficiently understood. In particular, few studies have systematically clarified how RFA content influences the coupled evolution of workability, mechanical performance, durability, and microstructure in such systems. Moreover, although the durability of cement-based FSS has been preliminarily investigated [26], systematic evidence regarding the durability of RFA-containing all-solid-waste-based FSS under wetting–drying and freeze–thaw cycles remains limited.

To address these issues, this study developed an all-solid-waste-based FSS using soft clay and RFA as the primary raw materials, together with a solid-waste-based binder composed of GGBS, CS, and DG, while FA was incorporated to improve workability. The effects of different RFA contents (0%, 20%, 40%, 60%, and 80%) on workability, mechanical properties, and durability under wetting–drying and freeze–thaw cycles were systematically investigated. In addition, XRD and SEM were employed to characterize the microstructural features associated with the performance evolution of the mixtures with different RFA contents. Rather than evaluating RFA solely in terms of flowability or strength, this study assessed its role through an integrated framework that jointly considered fresh-state behavior, mechanical performance, durability, and microstructural characteristics. The findings are expected to provide a useful reference for the mixture design and performance optimization of RFA-containing FSS, and to further enhance the understanding of high-value solid-waste utilization in sustainable geotechnical materials.

## 2. Materials and Methods

### 2.1. Raw Materials

#### 2.1.1. Soft Clay

**Figure 1:**
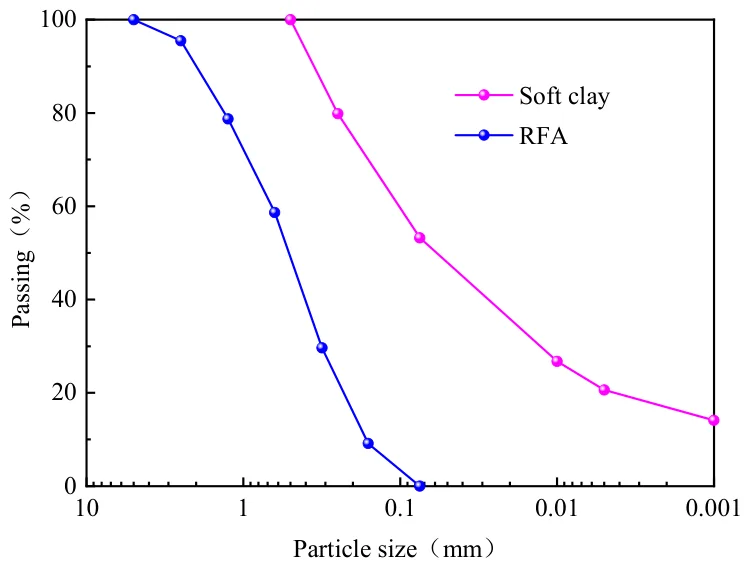
Particle size distribution.

The soft clay used in this study was collected from foundation pit excavation at a construction site in Qixia District, Nanjing, China. After sampling, the soil was oven-dried at 110 °C for 24 h to remove moisture, and then crushed and ground into dry clay powder for subsequent use. Its basic geotechnical properties were determined in accordance with the Chinese standard JTG 3430 [27]. The particle size distribution and main physical properties of the soft clay are shown in Figure 1 and Table 1, respectively.

#### 2.1.2. Recycled Fine Aggregate

The recycled fine aggregate (RFA) was obtained from waste concrete after crushing, washing, and screening at a local construction waste recycling plant in Nanjing, China. The particle size distribution curve of the RFA, determined by sieve analysis, is shown in Figure 1. The results indicate that the material had a particle size range primarily between 0.15 and 2.5 mm, which is suitable for use in a flowable slurry system. The RFA particles exhibited pronounced angularity and rough surfaces, with a morphology intermediate between crushed stone and rounded gravel. The water absorption and apparent density of the RFA were 9.7% and 2210 kg/m^3^, respectively, meeting the relevant requirements of the Chinese standard GB/T 25176 [28]. In addition, the crushing value of the RFA was 16.5%, the adhered mortar content was 13.8%, and the mud content was lower than 1.0% by mass. Because recycled aggregates are inherently source-dependent, the most suitable RFA content identified in this study should be interpreted with reference to the specific source and quality of the RFA used herein, rather than as a universal value.

#### 2.1.3. Solid-Waste-Based Binder

The solid-waste-based binder consisted of GGBS, CS, and DG. The GGBS was S95-grade slag powder supplied by Jiangsu Rongda New Materials Co., Ltd. (Nantong, China). It had a specific surface area of approximately 400–600 m^2^/kg, and its particle sizes were mainly distributed in the range of 10–40 μm. The CS was collected from a chemical plant in Changzhou, China. It mainly consisted of Ca(OH)_2_ and exhibited a fine powder morphology, with particle sizes mainly distributed in the range of 5–50 μm. The DG was obtained from a coal-fired power plant in Nanjing, China. Its main component was CaSO_4_·2H_2_O, and its particle sizes were mainly distributed in the range of 20–60 μm. The chemical compositions of the above three solid-waste materials, as analyzed by X-ray fluorescence spectrometry, are summarized in Table 2.

#### 2.1.4. Fly Ash

Fly ash (FA) was also incorporated into the FSS to improve its workability [29]. The FA was purchased from Jiangsu Rongda New Materials Co., Ltd. It consisted mainly of spherical particles and had a specific surface area of approximately 300–600 m^2^/kg. Its particle sizes were primarily distributed between 15 and 60 μm. Its chemical composition is summarized in Table 2.

#### 2.1.5. Superplasticizer

A polycarboxylate-based high-range water reducer was used to regulate the properties of the fresh FSS slurry. Its water-reducing rate exceeded 25%. The superplasticizer was incorporated to improve the dispersion of solid particles and enhance the flowability of the slurry at the selected water-to-solid ratio.

### 2.2. Preparation Methods

#### 2.2.1. Mixture Design

The all-solid-waste-based FSS was prepared using soft clay, RFA, a solid-waste-based binder, FA, superplasticizer, and water. Based on preliminary experiments and previous studies [20,30], the mass ratio of GGBS:CS:DG in the solid-waste-based binder was fixed at 72:20:8. To evaluate the effect of RFA content, soft clay was partially replaced by RFA on an equal-mass basis. The RFA content, defined as the mass percentage of RFA in the combined mass of RFA and dry clay, was set at 0%, 20%, 40%, 60%, and 80%. The corresponding mixtures were designated as R0, R20, R40, R60, and R80, respectively. These replacement levels were selected to cover a broad yet practical range from clay-dominant to aggregate-dominant compositions. A uniform interval of 20% was adopted to facilitate trend identification and optimum screening while maintaining experimental comparability. The upper limit of 80% was chosen to represent a high-substitution condition while preserving specimen formability and matrix continuity.

For all mixtures, the contents of the solid-waste-based binder and fly ash (FA) were fixed at 9% and 6%, respectively, based on the total mass of soft clay, RFA, binder, and FA. The superplasticizer content was fixed at 3.12 kg/m^3^ according to the total volume of fresh FSS slurry. In addition, the water-to-solid ratio was maintained at 0.32, defined as the mass ratio of water to the total mass of soft clay, RFA, binder, and FA. The detailed mix proportions of the all-solid-waste-based FSS are presented in Table 3. These fixed parameters were selected on the basis of preliminary mixture design trials and engineering feasibility considerations to achieve adequate slurry flowability, acceptable setting characteristics, sufficient strength development at early and later curing ages, and overall mixture stability for FSS applications. To isolate the effect of RFA content, the binder content, FA content, water-to-solid ratio, and superplasticizer content were kept constant throughout the experimental program. Therefore, under the present experimental conditions, the variations observed in the fresh-state, mechanical, durability, and microstructural properties of the all-solid-waste-based FSS can be mainly attributed to differences in RFA content.

#### 2.2.2. Specimen Preparation

All dry materials, including clay powder, RFA, GGBS, CS, DG, and FA, were weighed according to the designed proportions. The dry materials were first mixed in a mortar mixer for at least 60 s to ensure uniformity. Subsequently, the required amounts of water and superplasticizer were added, and wet mixing was continued for 10 min until a homogeneous slurry was obtained.

**Figure 2:**
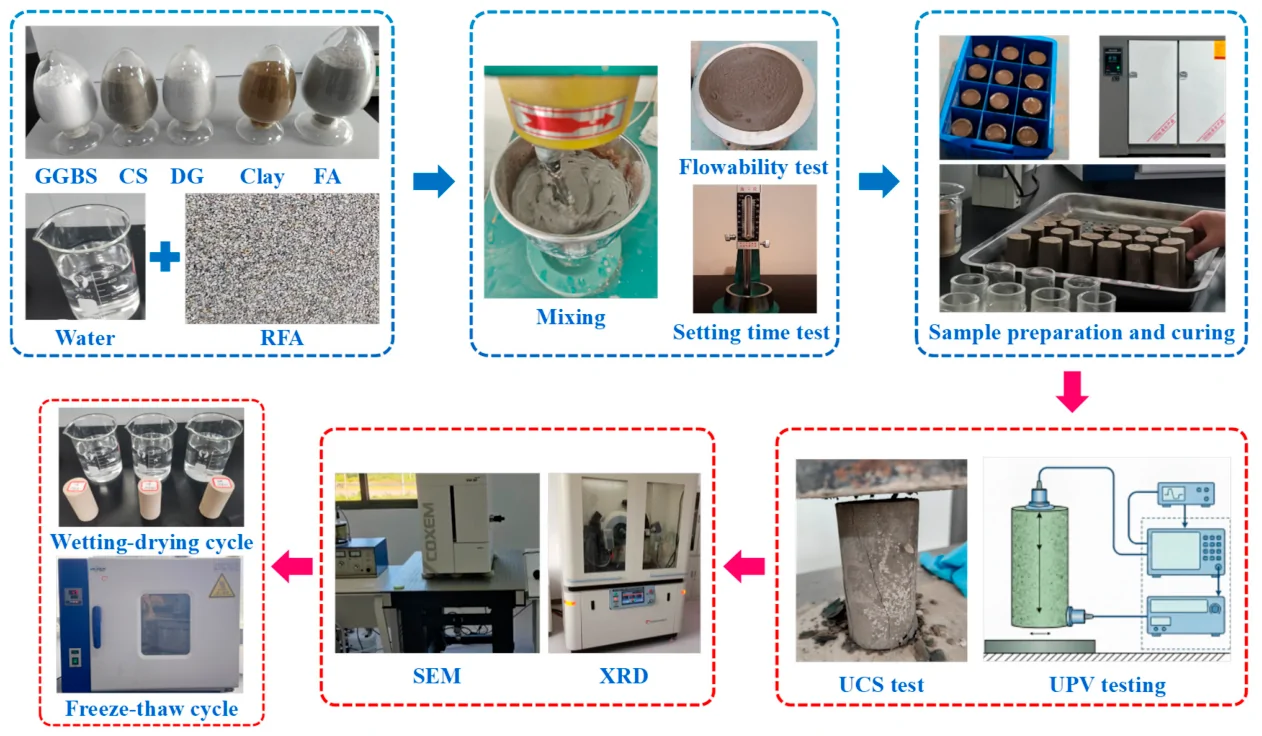
Specimen preparation and testing procedures.

Immediately after mixing, part of the fresh FSS slurry was used for flowability and setting time tests. The remaining slurry was then cast into cylindrical molds with a diameter of 39.1 mm and a height of 80 mm. After casting, the molds were stored in a standard curing chamber at (20 ± 2) °C and a relative humidity of no less than 95%. After 24 h, the specimens were demolded and returned to the curing chamber until the designated testing age. At the target curing age, the specimens were removed for mechanical tests, durability tests, and microstructural analyses. The specimen preparation and testing procedures are illustrated in Figure 2.

### 2.3. Test Methods

#### 2.3.1. Flowability and Setting Time

Flowability was evaluated using a cylinder spread test [16]. Fresh FSS slurry was poured into a smooth cylindrical mold with an inner diameter of 75 mm and a height of 150 mm. After the mold was lifted vertically, the slurry spread freely under its own weight. The maximum spread diameter and the diameter perpendicular to it were measured, and their mean value was taken as the flowability. Three parallel tests were conducted for each mixture, and the mean value was reported.

The initial and final setting times were determined using a Vicat apparatus in accordance with the Chinese standard GB/T 1346 [31]. This standard was adopted because the investigated FSS system contains a cementitious binder and exhibits setting characteristics that can be evaluated using a standard penetration-based method developed for cement paste-like materials. Under the present laboratory conditions, GB/T 1346 therefore provided an appropriate reference framework for the comparative assessment of setting behavior. The reported values are the means of three parallel measurements.

#### 2.3.2. UPV and UCS

The longitudinal ultrasonic pulse velocity (UPV) of cylindrical specimens was measured at curing ages of 3, 7, and 28 d using an ultrasonic detector. During testing, the transmitter and receiver were placed at the two ends of the specimen along its axial direction so that the ultrasonic wave propagated axially through the specimen. UPV is commonly used as an indirect indicator of internal compactness and defect distribution; a higher UPV generally indicates a denser and more continuous internal structure. The test procedure was carried out in accordance with the Chinese standard JGJ/T 411 [32].

After UPV measurement, specimens from the same batch were subjected to unconfined compressive strength (UCS) testing using a microcomputer-controlled universal testing machine. For each mixture and curing age, three parallel specimens were tested, and their mean values were used as representative results.

#### 2.3.3. Wetting–Drying and Freeze–Thaw Cycles

The wetting–drying cycle test was performed on specimens cured for 28 d. One wetting–drying cycle consisted of 24 h of natural drying at room temperature followed by 24 h of water immersion. After each cycle, the specimens were removed, surface water was wiped off, and the specimens were allowed to stand for 1 h before mass and UCS measurements were conducted. The numbers of cycles were set at 2, 4, 6, 8, 10, and 12. Specimens continuously maintained under standard curing conditions were used as the control group. The mass loss rate and UCS loss rate were determined by comparing the cycled specimens with the corresponding control specimens [33]. The mass loss rate and UCS loss rate were calculated as follows:

(1) $$M_{l}\;=\;\frac{M_{0}-M_{n}}{M_{0}}\times 100\%$$

(2) $$S_{l}=\frac{S_{0}-S_{n}}{S_{0}}\times 100\%$$

where *M_l_* and *S_l_* are the mass loss rate and UCS loss rate after *n* cycles, respectively; *M*_0_ and *M_n_* are the masses of the control specimen and the cycled specimen, respectively; and *S*_0_ and *S_n_* are the UCS values of the control specimen and the cycled specimen, respectively.

The freeze–thaw cycle test was also conducted on specimens cured for 28 d. One freeze–thaw cycle consisted of freezing at (−20 ± 2) °C for 12 h and thawing in clean water at (20 ± 2) °C for 12 h. After 2, 4, 6, 8, 10, and 12 cycles, the mass and UCS of the specimens were measured. Specimens kept under standard curing conditions were used as controls. The mass loss rate and UCS loss rate were calculated in the same manner as in the wetting–drying test. The adopted wetting–drying and freeze–thaw tests were intended to serve as accelerated laboratory indicators for comparative durability evaluation among mixtures, rather than as direct predictors of field service life.

#### 2.3.4. XRD and SEM Analyses

XRD analysis was performed on representative specimens cured for 28 d using a TD-3500 diffractometer (Dandong Tongda, Dandong, China) with Cu-Kα radiation (λ = 0.154 nm) operating at 40 kV. The scanning range was set to 5–85° (2*θ*) with a step size of 0.02° and a dwell time of 0.2 s per step. SEM imaging was conducted using a COXEM EM-30 microscope (COXEM Co., Ltd., Daejeon, Republic of Korea) at an accelerating voltage of 15 kV and a magnification of 5000× to visualize hydration product morphology, particle bonding, and pore-related structural features. The working distance was approximately 15 mm. Samples were oven-dried to constant weight and coated with a thin gold layer before analysis. Three fields were imaged per specimen, with selected fields determined based on the presence of visible hydration products and representative microstructural features, deliberately avoiding mechanical defects introduced during specimen preparation.

The XRD and SEM results were used to support the interpretation of hydration-product evolution and overall structural compactness. Direct quantitative characterization of the pore structure was not included in the present study. Therefore, the SEM observations were used as qualitative microstructural evidence rather than as a basis for quantitative pore-structure analysis.

#### 2.3.5. Data Treatment

Unless otherwise stated, the reported results are presented as the mean values of three parallel specimens, and the corresponding standard deviations (SDs) are provided in the figures, where applicable, to reflect data variability. In addition, although significance testing is generally important in the experimental investigation of cementitious materials [34], the parallel specimens in this study represent technical replicates rather than independent experimental replications. Therefore, the data were used primarily to identify comparative trends among mixtures rather than to support rigorous statistical inference. Accordingly, formal significance testing was not performed, so as to avoid overinterpretation of results derived from data with limited statistical power.

## 3. Results and Analyses

### 3.1. Flowability and Setting Time

**Figure 3:**
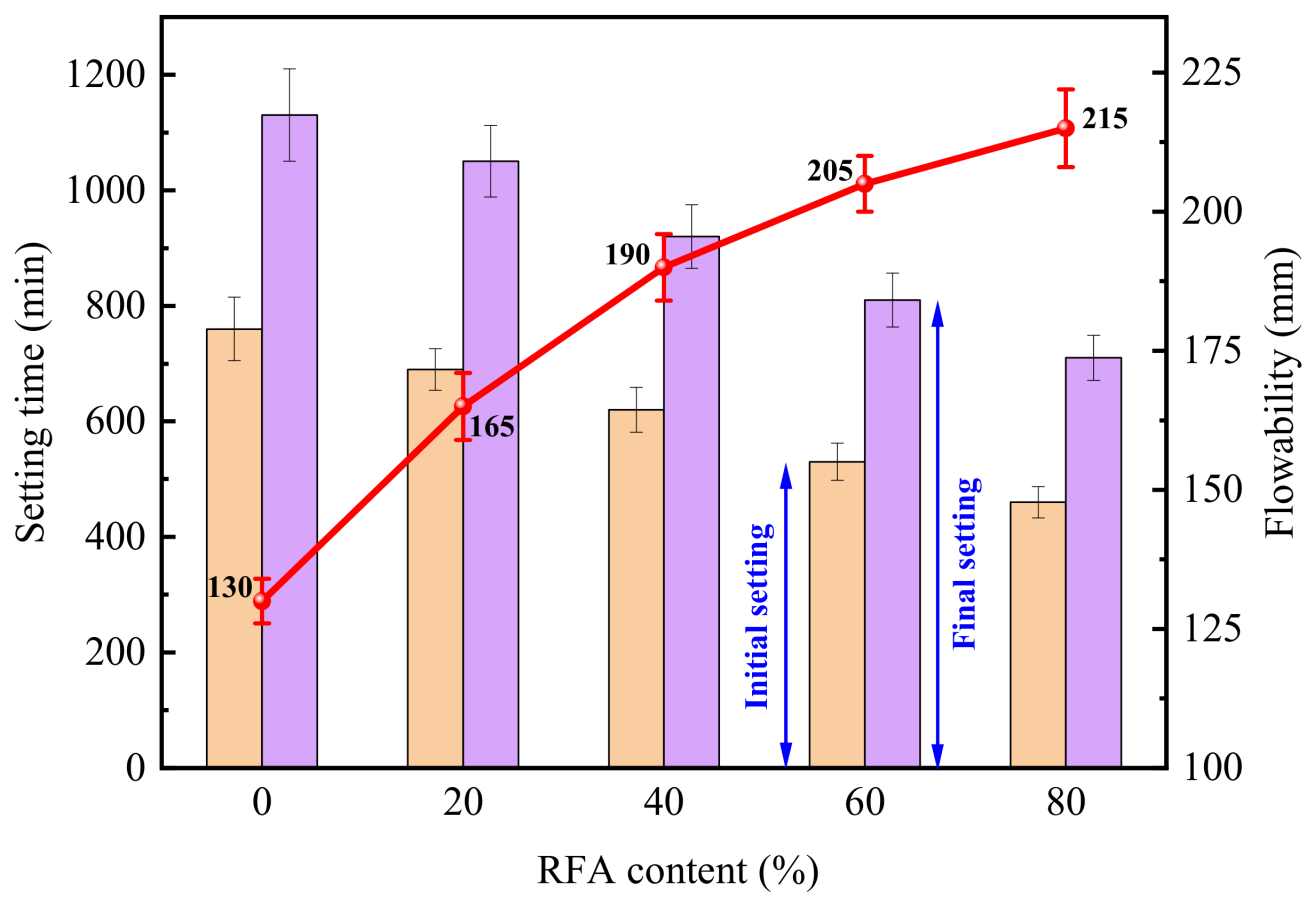
Effect of RFA content on the flowability and setting time of the FSS slurry. Error bars represent SD. The orange columns indicate the initial setting time, and the purple columns indicate the final setting time.

Figure 3 presents the effects of RFA content on the flowability and setting time of the FSS slurry. As the RFA content increased, the flowability increased continuously. The flowability of the control group R0 was 130 mm. When the RFA content increased to 40% and 80%, the flowability increased to 190 mm and 215 mm, corresponding to increases of 46.2% and 65.4%, respectively.

This trend can be attributed primarily to the different water-demand and interaction characteristics of soft clay and RFA. Soft clay contains a large proportion of very fine particles and clay minerals with strong water absorption and surface adsorption capacities. In clay-rich mixtures, a considerable amount of mixing water is therefore bound to particle surfaces, reducing the free water available for slurry flow. In addition, fine clay particles tend to agglomerate and may coat binder and FA particles, further hindering dispersion. By contrast, RFA particles are coarser and less cohesive. Replacing part of the clay with RFA therefore reduces the amount of bound water and improves particle mobility, leading to higher flowability.

The setting behavior changed in a similar manner. Both the initial and final setting times decreased with increasing RFA content. In the control mixture, the high clay content delayed hydration and structure formation because clay minerals adsorbed Ca^2+^ and early hydration products, thereby restricting the nucleation and growth of cementitious phases. Partial replacement of clay with RFA weakened this retardation effect and allowed the solid-waste-based binder to react more effectively, thereby accelerating setting.

These results show that RFA can improve fresh-state performance by simultaneously enhancing flowability and shortening the setting time. However, the appropriate RFA content should be determined through a combined assessment of fresh-state behavior, mechanical performance, and durability rather than flowability alone.

### 3.2. UCS and UPV

**Figure 4:**
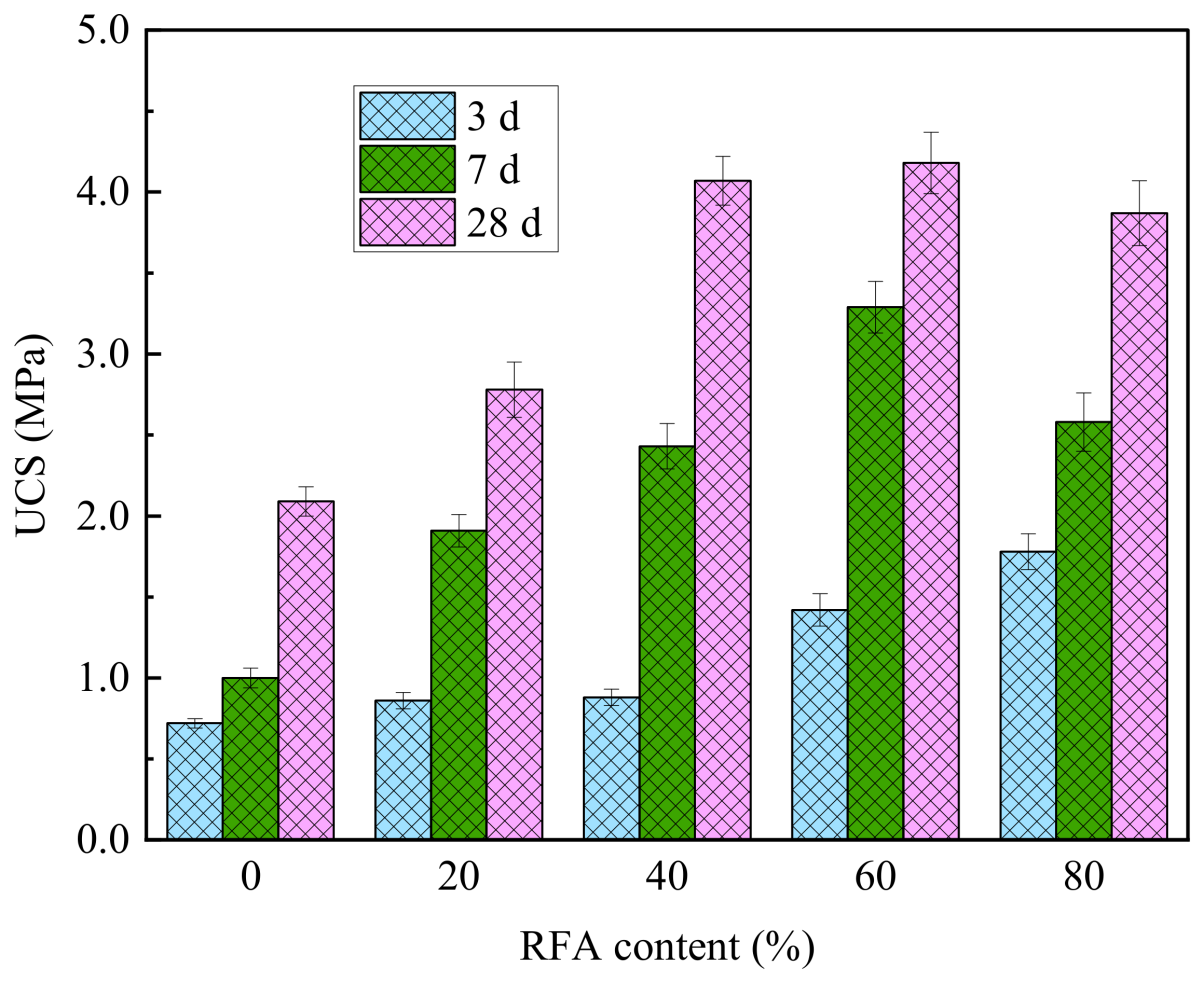
Variation in the UCS of FSS with different RFA contents. Error bars represent SD.

**Figure 5:**
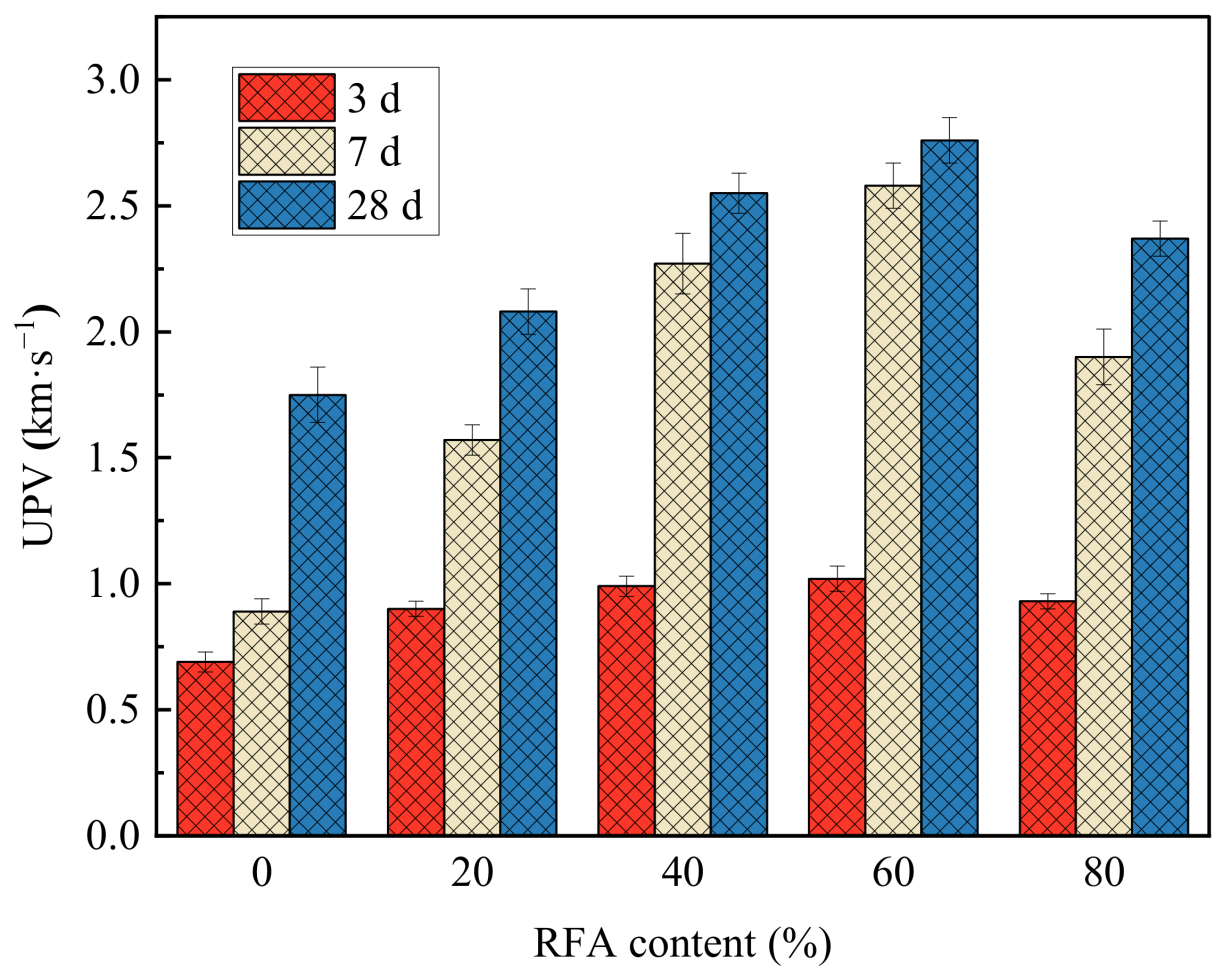
Variation in UPV of FSS with different RFA contents. Error bars represent SD.

Figure 4 and Figure 5 show the variations in UCS and UPV of FSS with different RFA contents at curing ages of 3, 7, and 28 d. The numerical mean ± SD values for UCS and UPV are provided in Table 4. With increasing curing age, the UCS of all specimens increased considerably, and the UPV increased accordingly. This is mainly because more hydration products were generated as curing proceeded, which continuously enhanced particle bonding and increased structural compactness, thereby improving both UCS and UPV.

Overall, both UCS and UPV first increased and then decreased with increasing RFA content. At 40% RFA, the 28 d UCS and UPV were 94.7% and 45.5% higher, respectively, than those of the specimen without RFA. When the RFA content increased to 60%, both indices remained at relatively high levels, whereas a further increase to 80% caused both values to decline.

This behavior can be interpreted as the combined result of particle skeleton formation, gradation optimization, and possible secondary hydration associated with adhered mortar on the RFA surface. Because RFA has relatively high strength and hardness, it can serve as a stable skeleton within the FSS matrix. At moderate content, it also improves particle packing and increases structural compactness. In addition, the residual mortar attached to the RFA surface may undergo further hydration under alkaline conditions, thereby strengthening interparticle bonding. When the RFA content becomes too high, however, the particle system becomes less balanced and tends to contain more large voids. Under such conditions, the available hydration products and soft clay particles are insufficient to fill the enlarged void space effectively, resulting in reduced compactness and lower strength. This interpretation is consistent with the decline in both UCS and UPV at 80% RFA.

From the perspective of mechanical performance, the favorable RFA content range for the present FSS system was therefore 40–60%.

### 3.3. Resistance to Wetting–Drying Cycles

**Figure 6:**
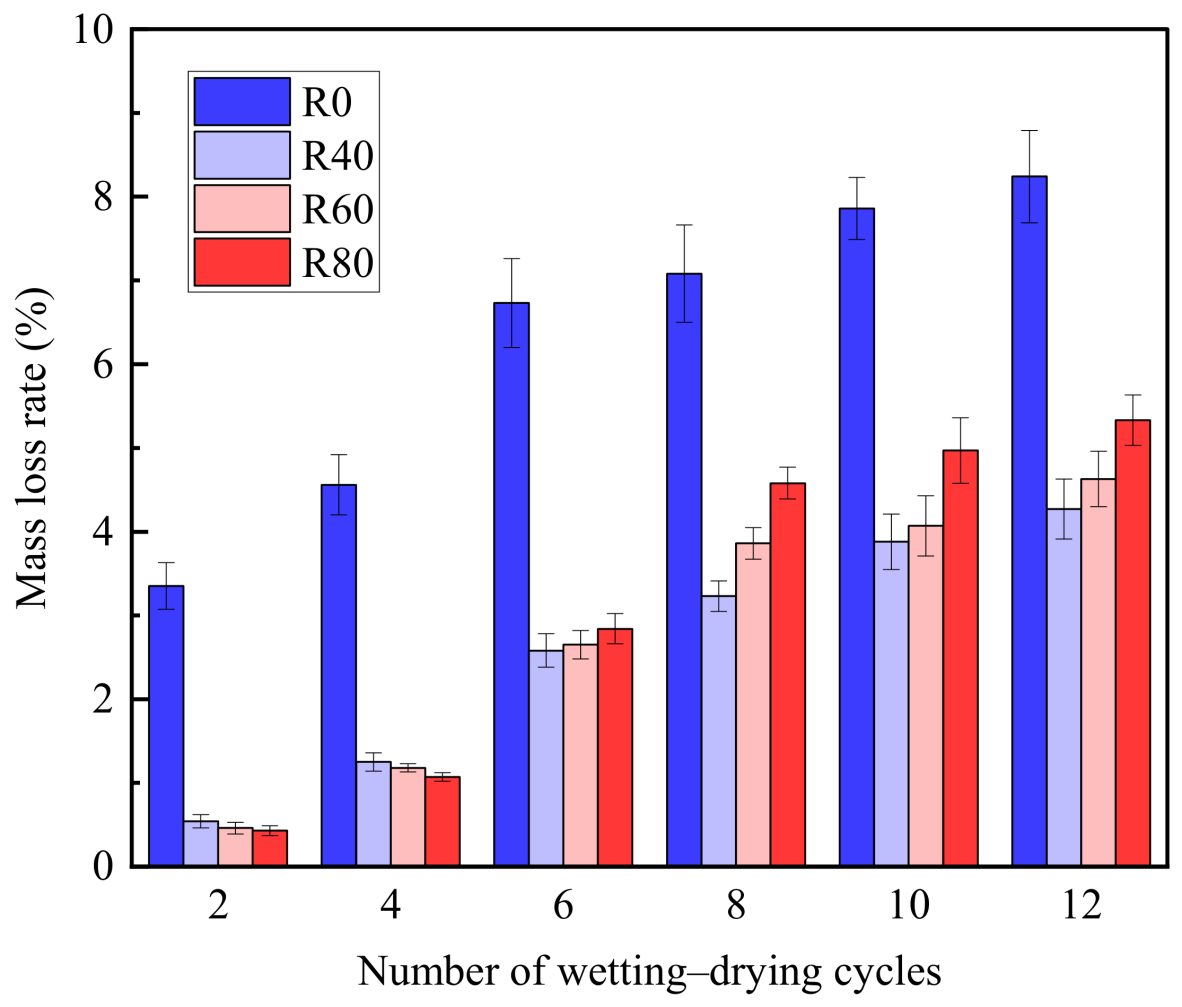
Mass loss rates of the specimens subjected to wetting–drying cycles. Error bars represent SD.

Figure 6 shows the mass loss rates of the specimens after 2–12 wetting–drying cycles, with the specific numerical results (mean ± SD) provided in Table 5. For all specimens, the mass loss rate increased with the number of cycles, reflecting progressive surface erosion and internal damage caused by repeated shrinkage–swelling actions. The increase was faster in the early cycles and gradually stabilized thereafter. During the initial cycles, repeated drying shrinkage and wetting expansion triggered by moisture migration caused the weakly bonded surface regions to peel off more easily, leading to rapid mass loss. As the most vulnerable surface materials were removed, the remaining internal structure became more stable, resulting in a slower rate of mass loss in subsequent cycles.

Among all specimens, the control specimen R0 exhibited the highest mass loss, reaching 8.24% after 12 cycles. In contrast, the mass loss rates of R40, R60, and R80 after 12 cycles were only 4.27%, 4.63%, and 5.33%, respectively. These results indicate that the incorporation of RFA improves the resistance of FSS to wetting–drying deterioration under the present test conditions.

**Figure 7:**
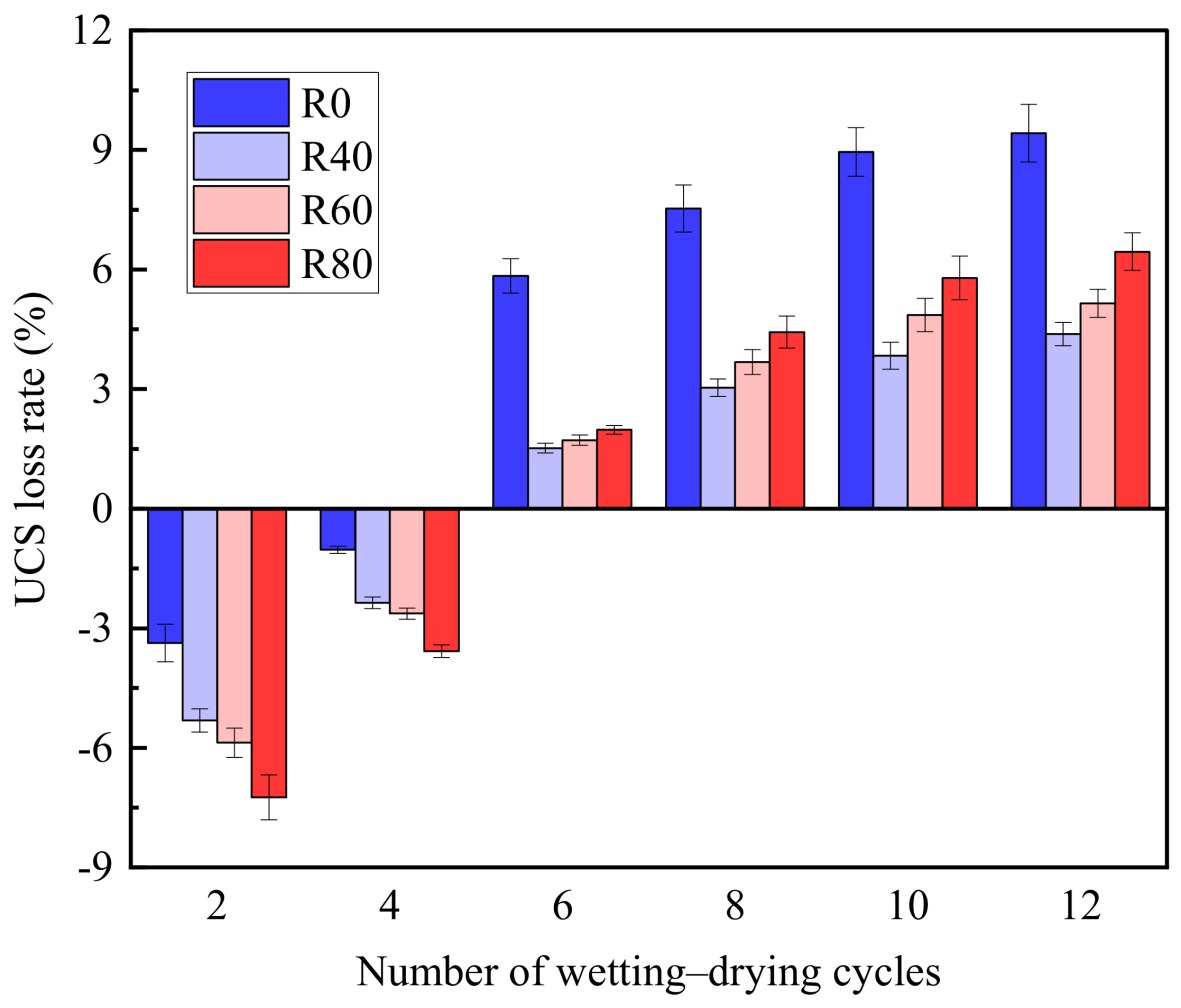
UCS loss rates of specimens subjected to wetting–drying cycles. Error bars represent SD.

The UCS loss behavior is illustrated in Figure 7, with the corresponding numerical results (mean ± SD) presented in Table 5. A notable feature is that the UCS loss rate was negative during the early stage for all specimens, especially within the first four cycles. This indicates that the cycled specimens temporarily exhibited slightly higher strength than the control specimens. The phenomenon can be explained by hydration compensation. During the wetting stage, water re-entered the specimens and promoted further hydration of previously unreacted or partially reacted binder components. The newly formed hydration products partially filled internal pores and temporarily offset the mechanical deterioration caused by repeated wetting–drying action. As the number of cycles increased, however, this compensating effect gradually weakened, whereas cyclic damage became dominant. From approximately the sixth cycle onward, the UCS loss rate became positive and then continued to increase. After 12 cycles, the control specimen exhibited a UCS loss rate of 9.42%, whereas the R40, R60, and R80 groups showed much lower values of 4.38%, 5.15%, and 6.45%, respectively.

The improved wetting–drying resistance of FSS containing a moderate amount of RFA can be attributed to the combined effects of skeleton formation and microstructural compactness. The relatively stable particle skeleton formed by RFA restrained deformation caused by moisture variation, while the denser matrix hindered water penetration and reduced crack propagation. Among the investigated mixtures, R40 showed the lowest mass loss and UCS loss after repeated wetting–drying cycles. Therefore, under the present experimental conditions, 40% RFA appeared to be the most favorable content for wetting–drying resistance.

### 3.4. Resistance to Freeze–Thaw Cycles

**Figure 8:**
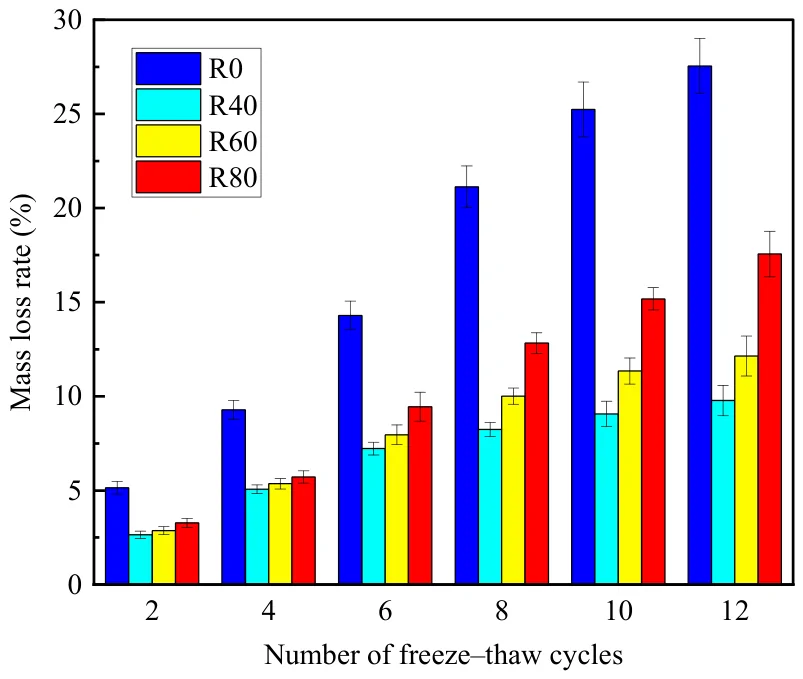
Mass loss rates of the specimens subjected to freeze–thaw cycles. Error bars represent SD.

**Figure 9:**
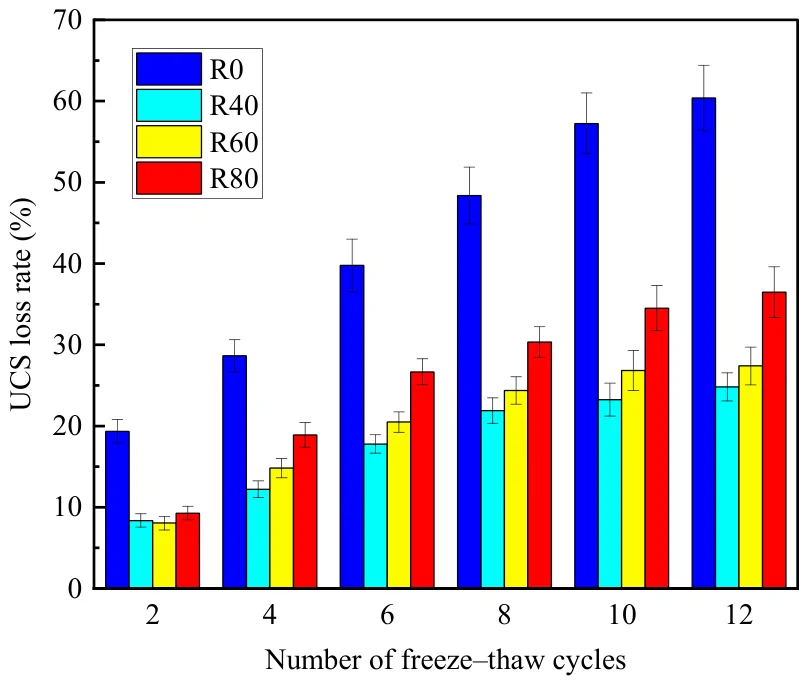
UCS loss rates of specimens subjected to freeze–thaw cycles. Error bars represent SD.

The mass loss and UCS loss of the specimens after freeze–thaw cycling are depicted in Figure 8 and Figure 9, respectively. The specific numerical values for the mass and UCS loss rates (mean ± SD) are detailed in Table 6. Compared with wetting–drying action, freeze–thaw cycling causes much more severe deterioration. This is because the repeated freezing and thawing of pore water generated substantial expansion–contraction stresses within the material matrix, thereby accelerating crack propagation and surface damage [35].

For all specimens, both mass loss and UCS loss increased continuously with the number of freeze–thaw cycles. The control group R0 exhibited the poorest resistance. After 12 cycles, its mass loss rate and UCS loss rate reached 27.55% and 59.97%, respectively, indicating severe frost damage. The incorporation of RFA markedly improved freeze–thaw resistance, suggesting that RFA enhanced the structural integrity of FSS and mitigated freeze–thaw-induced deterioration. Among all specimens, R40 showed the best overall performance. After 12 cycles, its mass loss rate and UCS loss rate were only 9.78% and 24.82%, respectively, which were much lower than those of the control specimen. Although R60 and R80 still performed better than R0, their freeze–thaw resistance was inferior to that of R40.

The improved freeze–thaw resistance at a moderate RFA content was closely related to microstructural refinement. Moderate RFA incorporation promoted a denser matrix with a less connected pore structure, thereby reducing the amount of freezable water and limiting the space available for ice expansion. Excessive RFA, however, increased the proportion of larger pores and weakened the beneficial structural role of RFA during freeze–thaw cycling, resulting in greater mass and strength losses. Accordingly, 40% RFA was the most favorable content for freeze–thaw resistance under the conditions examined in this study.

### 3.5. XRD Analysis

**Figure 10:**
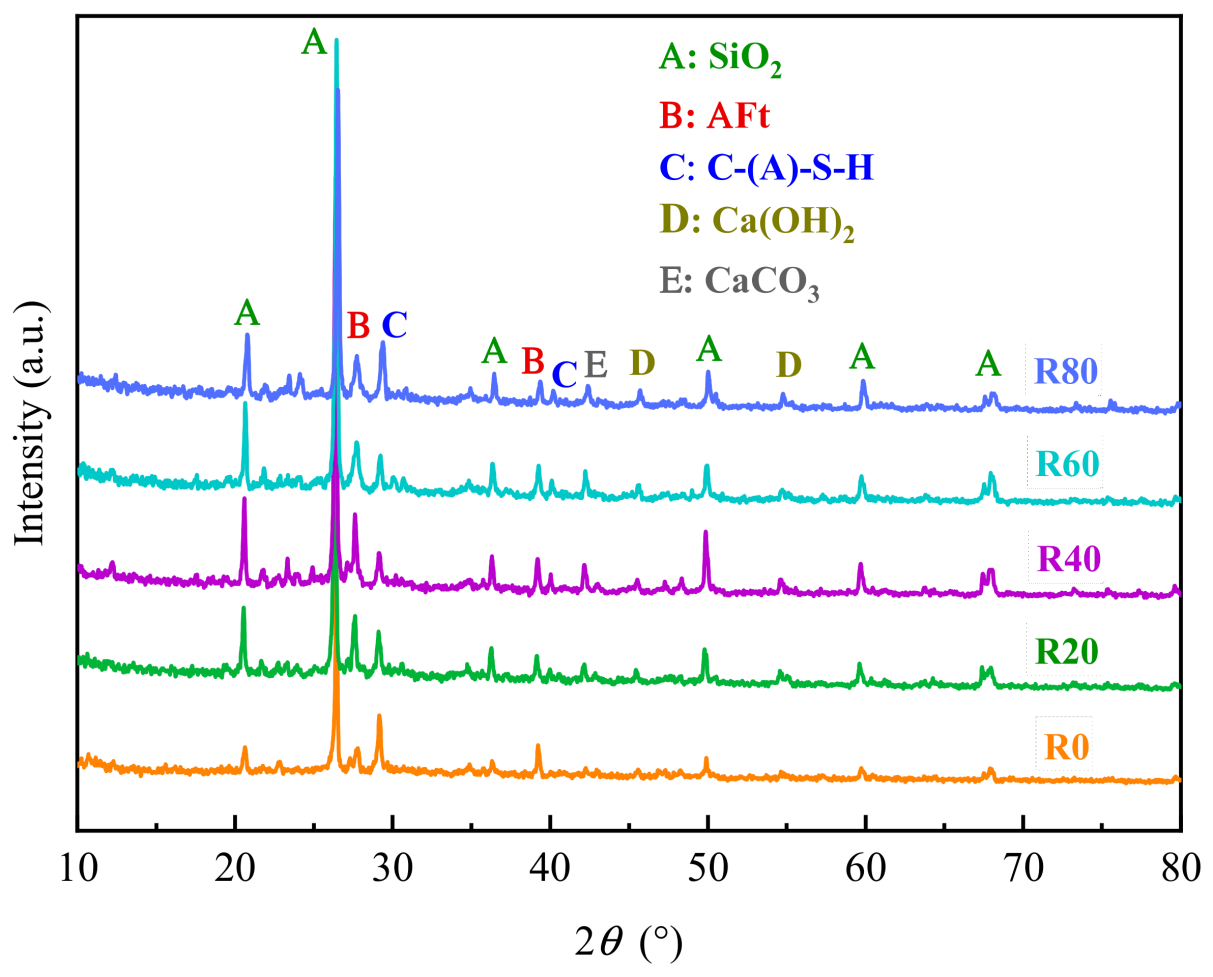
XRD analysis.

The XRD patterns of the 28 d specimens are presented in Figure 10. Although the peak intensities varied among the groups, the major crystalline phases were generally similar in all specimens. This indicates that the incorporation of RFA did not fundamentally alter the hydration mechanism of the solid-waste-based binder system. Instead, RFA mainly affected the amount, distribution, and microstructural effectiveness of the hydration products by modifying the physical environment of the reaction system.

In this FSS system, GGBS was activated under the alkaline conditions provided by CS, releasing reactive silicate and aluminate species, which subsequently reacted with Ca^2+^ to generate C-(A)-S-H-gel-related phases [30]. Meanwhile, sulfate supplied by DG promoted AFt formation through its reaction with GGBS and CS [20]. No obvious residual CaSO_4_ peak was detected, indicating that sulfate actively participated in the hydration process. These hydration products bonded the particles together and filled internal pores, thereby contributing to strength development and structural compactness.

Overall, the XRD results indicate that the main hydration products in the FSS system were C-(A)-S-H-gel-related phases and AFt. Together with the macroscopic results, these products were considered to contribute to the development of strength and durability. However, the XRD analysis in this study was qualitative and used mainly for phase identification. No relative intensity comparison, peak-area quantification, or Rietveld refinement was performed. Therefore, the XRD results were interpreted cautiously and used only as qualitative support for the phase-related discussion.

### 3.6. SEM Analysis

**Figure 11:**
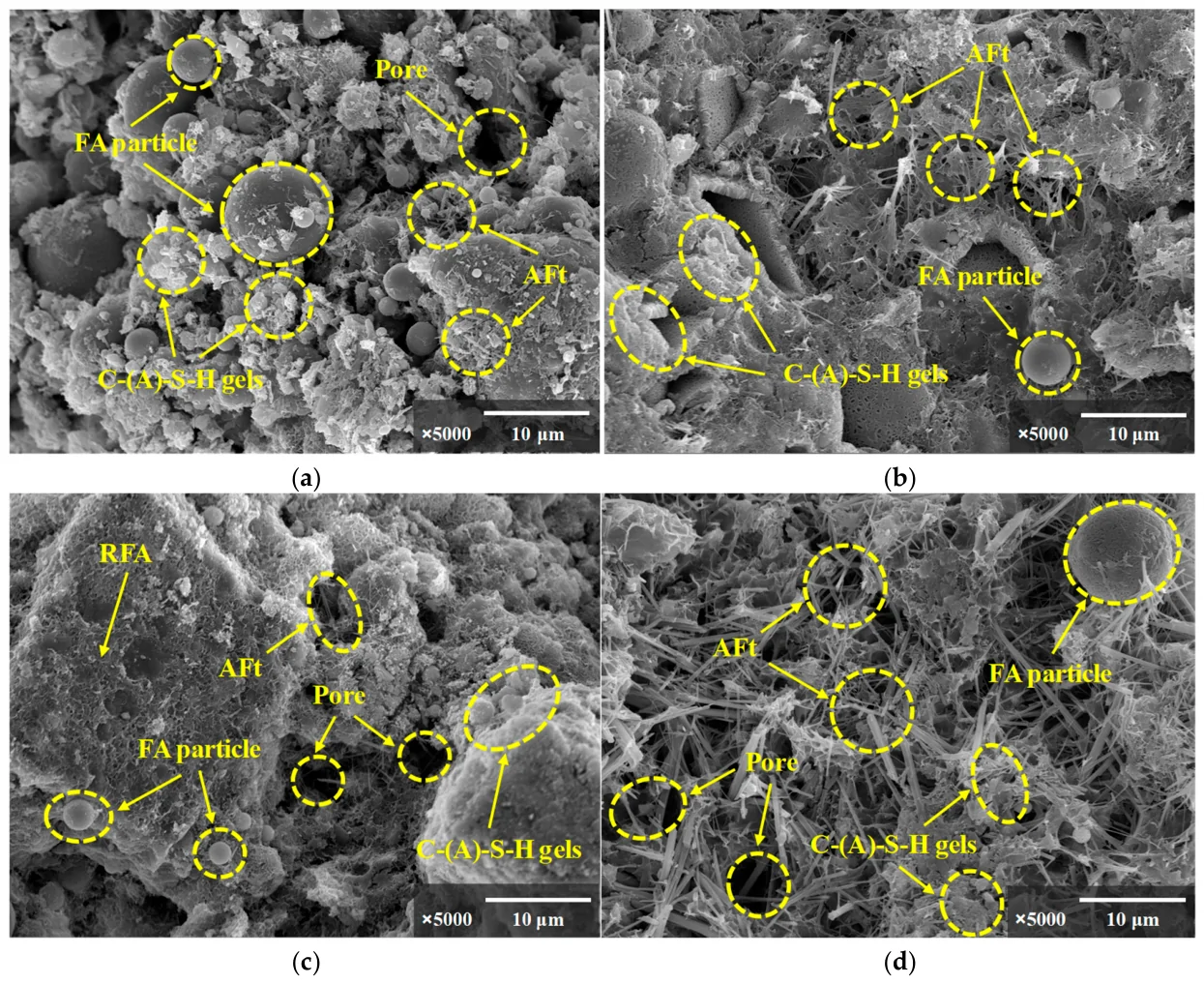
SEM analysis: (**a**) R0; (**b**) R40; (**c**) R60; (**d**) R80.

SEM images of representative 28 d specimens are shown in Figure 11. Some differences in local microstructural features can be observed among the specimens. In all groups, hydration products mainly appeared as gel-like masses and needle-like or rod-like crystals. In addition, some spherical FA particles remained visible in the matrix.

The control specimen R0 showed a relatively looser microstructural appearance in the selected field, with fewer visible hydration products and more apparent pores. This observation is generally consistent with its lower UCS and UPV, as well as its comparatively poorer durability. A possible explanation is that the high clay content hindered particle dispersion and hydration reactions, thereby limiting the formation of an effective binding network. By contrast, the specimen containing 40% RFA exhibited a comparatively denser microstructural appearance in the observed field. More hydration products, including C-(A)-S-H-gel-related phases and AFt crystals, were observed, and these phases appeared to interweave and connect surrounding particles. Large pores were less evident in this specimen, suggesting a relatively higher degree of pore filling and compactness in the local area observed.

When the RFA content was further increased to 60% and 80%, hydration products were still visible in considerable amounts; however, pores were also observed in the selected fields. In particular, the selected SEM image of R80 suggests relatively looser local microstructural features in the observed field. This may suggest that the performance decline at excessive RFA contents was related not to the absence of hydration products, but rather to less effective pore filling and reduced matrix continuity. Nevertheless, because the SEM analysis was based on a limited field of view, these observations are qualitative only and should not be interpreted as statistically representative of the overall microstructure of the specimens.

Overall, the SEM observations qualitatively support the macroscopic test results, suggesting that the suitable RFA content was associated with a balance between skeleton formation and pore filling. An appropriate amount of RFA appeared to favor the development of a relatively denser local microstructure, whereas excessive RFA may be associated with more apparent pores and lower local compactness.

## 4. Discussion

Flowability is one of the key performance indicators of FSS, and the required level depends on the intended engineering application. Previous studies have suggested that a flowability above 150 mm is generally required for practical use, and in some cases values above 180 mm are preferred [16,36]. In the present study, the specimen without RFA showed a flowability below 150 mm and would therefore have limited practical applicability. By contrast, the incorporation of RFA increased the flowability to above 180 mm, satisfying the requirements of most engineering scenarios. It should also be noted that the water-to-solid ratio is the most direct parameter controlling FSS flowability [9]. Although increasing the water-to-solid ratio can significantly enhance flowability, it may also adversely affect the strength and compactness of the hardened material. In this context, the incorporation of RFA provides an alternative means of improving fresh-state properties without relying solely on additional water, while still maintaining relatively favorable mechanical performance within an appropriate content range. Meanwhile, the reduction in setting time caused by RFA incorporation may be advantageous for improving construction efficiency, provided that adequate workable time is retained for transportation, placement, and finishing. For engineering projects requiring a longer construction window, the content of superplasticizer, FA, or mixing water should be appropriately adjusted in coordination with the selected RFA content.

The results further showed that the effect of RFA on the mechanical performance and durability of all-solid-waste-based FSS was not monotonic. Under the mixture conditions adopted in this study, both 40% and 60% RFA were favorable in terms of mechanical performance, whereas 40% RFA provided the best durability and the most balanced overall performance. These findings indicate that the most suitable RFA content should be determined through multi-indicator evaluation rather than from any single property. For the material system investigated here, 40% RFA is therefore considered the most appropriate content within the tested range.

Importantly, this optimum range is expected to depend strongly on the intrinsic properties of the RFA, including particle gradation, water absorption, particle morphology, crushing value, and adhered mortar content. Because RFA quality varies considerably with source [37], the optimum content identified in this study should not be generalized as a universal value. For example, the amount of adhered mortar affects not only the potential for secondary hydration but also the strength development and pore structure of the resulting FSS. Accordingly, establishing mixture design criteria based on key RFA quality indices should be an important focus of future research.

The type of raw soil is another important factor controlling the effectiveness of RFA. In this study, the raw soil was a soft clay with a high specific surface area and strong water adsorption capacity. Its strong ion adsorption and hydration-retarding effects clearly restricted slurry workability. Introducing RFA partially replaced the clay fraction, released more free water, and weakened ion adsorption, thereby improving both the flowability and the setting behavior. If the raw soil was instead a low-plasticity silt or sandy soil, the adverse effects associated with clay minerals would be much less pronounced, and both the improvement mechanism and the optimum RFA content would likely change. This suggests that FSS mixture design should be adapted to the characteristics of the raw soil in order to achieve coordinated optimization of workability, mechanical performance, and durability. In this sense, the present study provides a performance-based optimization framework rather than a fixed content rule.

The macroscopic performance trends were generally consistent with the microstructural observations. At moderate RFA contents, the increases in UCS and UPV could be attributed to the combined effects of a more favorable particle skeleton and a denser binding matrix, as evidenced by the SEM results. The XRD patterns further confirmed the presence of hydration products, including C-(A)-S-H-gel-related phases and AFt, which contributed to interparticle bonding and pore filling. At excessively high RFA contents, however, SEM images showed a less continuous matrix, accompanied by more pores and loose regions, which may account for the deterioration in mechanical performance despite the continued presence of the major hydration products. Nevertheless, this interpretation should be treated with caution, since direct quantitative pore-structure characterization was not conducted in the present study.

From an engineering perspective, different applications impose different performance requirements on FSS [8,36]. In cold regions, freeze–thaw resistance is especially important. In the present study, an RFA content of 40% reduced the UCS loss rate after 12 freeze–thaw cycles to 24.82%, providing a useful reference for cold-region applications. For backfilling in confined spaces where very high flowability is required, a somewhat higher RFA content may still be considered. In such cases, however, the binder system or aggregate gradation should be further optimized to compensate for the possible reduction in mechanical performance and durability. Overall, the results indicate that the performance of FSS can be tailored to different engineering needs through an appropriate adjustment of RFA content. The proposed material therefore shows promise for trench backfilling, void filling, and other applications requiring self-compacting and pumpable materials. From a resource-utilization perspective, the combined use of RFA and industrial solid wastes may reduce the consumption of natural resources and promote the high-value utilization of multiple waste streams.

The present study has several limitations that should be acknowledged. First, the durability evaluation was limited to 12 wetting–drying and freeze–thaw cycles under laboratory conditions. Although these results are suitable for comparative assessment among mixtures, they should not be directly interpreted as indicators of field service life. Second, environmental durability, leaching behavior, carbonation resistance, and heavy-metal mobility were beyond the scope of this study. Third, direct quantitative pore-structure characterization was not conducted, and the microstructural interpretation therefore relied mainly on qualitative evidence from XRD and SEM analyses. Future studies should incorporate quantitative pore-structure characterization using methods such as adsorption analysis and image-based porosity evaluation. Further work is also needed to investigate long-term durability, environmental safety, source variability, and field-scale performance before large-scale engineering applications can be recommended.

## 5. Conclusions

This study systematically evaluated the effects of RFA content on the workability, mechanical properties, durability, and microstructure of all-solid-waste-based FSS. Based on the experimental results, the following conclusions can be drawn:

(1) Increasing the RFA content improved slurry flowability and shortened the setting time of the FSS. This effect was mainly attributed to the partial replacement of highly adsorptive soft clay by RFA, which increased the amount of free water and weakened the hydration-retarding effect of clay minerals.
(2) Both UCS and UPV showed an increase-then-decrease trend with increasing RFA content. From the perspective of mechanical performance, the favorable RFA content range for the investigated FSS system was 40–60%.
(3) An appropriate amount of RFA enhanced the resistance of FSS to both wetting–drying and freeze–thaw cycles. Among all mixtures, the specimen containing 40% RFA exhibited the lowest mass loss and UCS loss under both durability conditions, indicating the best overall durability within the investigated range.
(4) XRD and SEM analyses suggested that the main hydration products in the FSS system were C-(A)-S-H-gel-related phases and AFt. Moderate RFA content was observed to correspond to relatively denser localized microstructural features within the selected SEM fields, whereas excessive RFA appeared to increase localized pore prevalence and disrupt matrix continuity in these areas.
(5) Considering workability, mechanical performance, durability, and microstructural characteristics together, an RFA content of 40% is recommended as the most suitable proportion for the all-solid-waste-based FSS system investigated in this study under the adopted raw material sources and laboratory test conditions.

Overall, RFA can serve not only as a waste-derived substitute material but also as an effective performance-regulating component in all-solid-waste-based FSS, showing promising potential for backfilling applications. Nevertheless, the present conclusions are influenced by the source characteristics of the RFA and soft clay, the laboratory durability conditions adopted in this study, and the absence of direct quantitative pore-structure characterization and environmental safety assessment. Further work is still needed on source variability, long-term durability, environmental behavior, and field-scale validation.

## Figures and Tables

**Table 1:** Main physical parameters of the soft clay.

Natural Water Content (%)	Maximum Dry Density (g·cm^−3^)	Void Ratio	Liquid Limit (%)	Plastic Limit (%)	Plasticity Index	Clay Content (%)	Organic Matter Content (%)	pH
28.6	1.78	1.15	30.7	12.4	18.3	45.3	3.63	6.5

**Table 2:** Chemical compositions of the solid-waste materials.

Materials	SiO_2_	Al_2_O_3_	CaO	Fe_2_O_3_	MgO	TiO_2_	P_2_O_5_	MnO_2_	SO_3_	Na_2_O	LOI
GGBS	32.21	11.16	38.45	1.43	10.28	1.87	–	0.85	–	–	2.75
CS	2.55	2.06	92.37	0.44	0.79	–	–	–	0.32	0.20	1.27
DG	0.96	1.17	45.39	–	0.34	–	0.62	–	42.20	1.37	7.95
FA	49.56	38.61	5.03	1.96	1.27	–	0.24	–	0.21	0.78	2.34

**Table 3:** Mix proportions of the all-solid-waste-based FSS/(kg/m^3^).

Test Name	Dry Clay	RFA	Solid-Waste-Based Binder			FA	Water	Superplasticizer
			GGBS	CS	DG			
R0	1105	0	84.2	23.4	9.4	78	416	3.12
R20	884	221	84.2	23.4	9.4	78	416	3.12
R40	663	442	84.2	23.4	9.4	78	416	3.12
R60	442	663	84.2	23.4	9.4	78	416	3.12
R80	221	884	84.2	23.4	9.4	78	416	3.12

**Table 4:** Numerical results of UCS and UPV for FSS specimens (mean ± SD).

RFA Content (%)	UCS (MPa)			UPV (km·s^−1^)		
	3 d	7 d	28 d	3 d	7 d	28 d
0	0.72 ± 0.03	1.00 ± 0.06	2.09 ± 0.09	0.69 ± 0.04	0.89 ± 0.05	1.75 ± 0.11
20	0.86 ± 0.05	1.91 ± 0.10	2.78 ± 0.17	0.90 ± 0.03	1.57 ± 0.06	2.08 ± 0.09
40	0.88 ± 0.05	2.43 ± 0.14	4.07 ± 0.15	0.99 ± 0.04	2.27 ± 0.12	2.55 ± 0.08
60	1.42 ± 0.10	3.29 ± 0.16	4.18 ± 0.19	1.02 ± 0.05	2.58 ± 0.09	2.76 ± 0.09
80	1.78 ± 0.11	2.58 ± 0.18	3.87 ± 0.20	0.93 ± 0.03	1.90 ± 0.11	2.37 ± 0.07

**Table 5:** Results of mass and UCS loss rates (mean ± SD) for FSS specimens under wetting–drying cycles.

Cycle Number	Mass Loss Rate (%)				UCS Loss Rate (%)			
	R0	R40	R60	R80	R0	R40	R60	R80
2	3.35 ± 0.28	0.54 ± 0.08	0.46 ± 0.07	0.43 ± 0.06	−3.37 ± 0.47	−5.31 ± 0.29	−5.87 ± 0.37	−7.24 ± 0.56
4	4.56 ± 0.36	1.25 ± 0.11	1.18 ± 0.05	1.07 ± 0.05	−1.03 ± 0.09	−2.36 ± 0.15	−2.63 ± 0.14	−3.57 ± 0.16
6	6.73 ± 0.53	2.58 ± 0.20	2.65 ± 0.17	2.84 ± 0.18	5.84 ± 0.43	1.52 ± 0.12	1.72 ± 0.13	1.98 ± 0.11
8	7.08 ± 0.58	3.23 ± 0.18	3.86 ± 0.19	4.58 ± 0.19	7.53 ± 0.59	3.04 ± 0.22	3.68 ± 0.31	4.43 ± 0.40
10	7.86 ± 0.37	3.88 ± 0.33	4.07 ± 0.36	4.97 ± 0.39	8.95 ± 0.61	3.84 ± 0.34	4.86 ± 0.42	5.79 ± 0.55
12	8.24 ± 0.55	4.27 ± 0.36	4.63 ± 0.33	5.33 ± 0.30	9.42 ± 0.72	4.38 ± 0.29	5.15 ± 0.35	6.45 ± 0.47

**Table 6:** Results of mass and UCS loss rates (mean ± SD) for FSS specimens subjected to freeze–thaw cycles.

Cycle Number	Mass Loss Rate (%)				UCS Loss Rate (%)			
	R0	R40	R60	R80	R0	R40	R60	R80
2	5.15 ± 0.34	2.65 ± 0.19	2.87 ± 0.21	3.28 ± 0.24	19.35 ± 1.46	8.36 ± 0.82	8.05 ± 0.84	9.27 ± 0.84
4	9.28 ± 0.51	5.07 ± 0.23	5.36 ± 0.28	5.72 ± 0.32	28.64 ± 1.98	12.22 ± 1.02	14.82 ± 1.20	18.90 ± 1.52
6	14.30 ± 0.76	7.23 ± 0.34	7.96 ± 0.52	9.45 ± 0.77	39.77 ± 3.25	17.78 ± 1.14	20.48 ± 1.26	26.67 ± 1.59
8	21.13 ± 1.11	8.25 ± 0.37	10.01 ± 0.43	12.83 ± 0.55	48.38 ± 3.51	21.89 ± 1.56	24.37 ± 1.68	30.32 ± 1.88
10	25.24 ± 1.45	9.07 ± 0.67	11.35 ± 0.69	15.18 ± 0.59	57.26 ± 3.76	23.25 ± 2.03	26.84 ± 2.47	34.51 ± 2.78
12	27.55 ± 1.47	9.78 ± 0.81	12.14 ± 1.06	17.56 ± 1.21	60.40 ± 4.01	24.82 ± 1.73	27.39 ± 2.33	36.48 ± 3.11

## Data Availability

The original contributions presented in this study are included in the article. Further inquiries can be directed to the corresponding author.
